# A Healthcare System’s Path to Carbon Neutrality: Addressing Climate Change and Role of the Health Care

**DOI:** 10.1016/j.focus.2025.100377

**Published:** 2025-06-06

**Authors:** Edward D. Shin, Rame Hemstreet, Stacey Alexeeff, Seema S. Wadwa, Jamal S. Rana

**Affiliations:** 1 Department of Medicine, Kaiser Permanente Northern California Medical Center, Oakland, California; 2 Kaiser Permanente, Oakland, Califronia; 3 Department of Cardiology, The Permanente Medical Group, Kaiser Permanente Oakland Medical Centre, Oakland, California; 4 Division of Research, Kaiser Permanente Northern California, Pleasanton, California

**Keywords:** Environmental determinants, social determinants, climate change, environmental health, environmental stewardship

## Abstract

**Introduction:**

Anthropogenic climate change, according to the WHO, results in approximately 150,000 deaths annually through mechanisms such as heat-related mortality, altered food production, and the spread of infectious diseases. With climate change predicted to cause over half a million climate-related deaths by 2050, healthcare systems, which contribute significantly to greenhouse gas emissions, must adopt roles in environmental stewardship.

**Methods:**

This descriptive case study details how Kaiser Permanente, a large nonprofit organization, became the first carbon-neutral healthcare system in the country.

**Results:**

Kaiser Permanente has demonstrated environmental stewardship through initiatives such as an on-site solar program, sustainability scorecards for suppliers, and extensive partnerships with organizations to support community health and environmental building efforts. Initiatives included scaling renewable energy usage, constructing Leadership in Energy and Environmental Design–certified facilities, and reducing water use intensity led to Kaiser Permanente being the first carbon-neutral health system in the country.

**Conclusions:**

The WHO has declared climate change as the most significant threat to human health. Kaiser Permanente’s journey to carbon neutrality highlights the critical role healthcare systems play in environmental stewardship. Continued focus on climate initiatives by the healthcare sector is essential to address the growing health impacts of climate change. Kaiser Permanente’s efforts provide a real-life and practical framework for achieving significant positive climate effects.

## HIGHLIGHTS

• Healthcare systems contribute 8% of global greenhouse gas emissions, doubling since 2010, yet only 10% of health companies have net zero targets.
• Kaiser Permanente achieved carbon neutrality by 2020 through renewable energy, Leadership in Energy and Environmental Design–certified hospitals, and supply chain reforms.
• Kaiser Permanente’s strategies ranging from onsite solar, policy advocacy, to supplier collaborations provide a scalable model for healthcare sustainability.

## INTRODUCTION

According to the WHO, anthropogenic climate changes have led to 150,000 deaths annually through heat-related mortality, changes in food production, and the spread of infectious disease.^1^ A study by Springmann et al.^2^ estimates that climate change could lead to 529,000 climate-related deaths worldwide by 2050 due to changes in dietary and weight-related risk factors. Paradoxically, healthcare systems represent significant drivers to climate change.^3^ Consequently, healthcare systems must consider adopting a more prominent role in environmental stewardship. A 2024 systematic review by Braithwaite and colleagues^4^ found how health systems could meaningfully reduce their environmental impact through several key approaches, including transitions to renewable energy, sustainable supply chains, and waste reduction strategies. For instance, the United Kingdom’s National Health Service has achieved significant progress through energy-efficient hospital designs.^4^ Similarly, in the U.S., institutions such as the Cleveland Clinic and Kaiser Permanente (KP) have emerged as leaders in sustainability through comprehensive initiatives, including solar investments.^4^ Despite these efforts, high upfront costs and fragmented state-level policies still present a challenge, thus underscoring the need for detailed case studies to identify scalable solutions.

Climate change worsens a person’s health through several mechanisms such as extreme temperatures disrupting regulatory pathways, dehydration, and premature activation of the renin–angiotensin system.^5^, ^6^, ^7^ In addition, climate change exacerbates respiratory disorders due to higher levels of air pollutants such as particulate matter, which can worsen pulmonary conditions.^8^ WHO estimates that 99% of the world’s population resides in places where air quality does not meet their standards, and a disproportionate proportion of the 4.2 million deaths worldwide in 2019 that can be attributed to air pollution occurs in low- and middle-income countries.^9^ Vector-borne diseases, such as malaria and dengue, are also influenced by changing climate patterns, altering the distribution and incidence of these diseases.^10^ In addition, extreme weather events can lead to physical injuries, heat-related illnesses, and disruptions in healthcare delivery.^10^ Notably, the real-time effects of climate change have already been experienced. In 2022, >1,700 deaths were due to heat-related causes, more than doubling over the past 5 years.^11^

According to the 2021 Intergovernmental Panel on Climate Change Report, greenhouse gases (GHGs) from human activities are responsible for the nearly 1.8°C warming in global temperatures since the 1850s.^12^ The last decade was the warmest on record, with a corresponding doubling in the prevalence of wildfires since 1984 and a nearly 40% reduction in the Artic ice thickness since the 1960s.^12^ Given current trajectories, the global average temperature is expected to warm at least twice as much in the next 100 years than during the prior century.^12^ Concerningly, the generated air pollution and projected climate change are expected to have disproportionate effects on marginalized communities, leading to a growing climate gap and a widening healthcare disparity.^13^ As such, alleviating this gap will require evaluation and actions along various pathways.

As previously mentioned, healthcare systems are major contributors to GHG emissions, contributing to nearly 8% of the current total global emissions, with doubling of such emissions from 2010 to 2018.^3^ Furthermore, the air pollution generated by healthcare system is estimated to be responsible for the loss of 388,000 disability-adjusted life-years in 2018.^14^ It is estimated that health systems spend over $8 billion each year on energy, accounting for nearly 10% of energy used by all commercial buildings.^3^ Despite such energy usage, according to a 2022 Accenture study of Global 2000 companies, health and life science sector companies combined have set the fewest net zero targets of all industries, with only 10% of health companies having net zero goals in place.^15^ As an example, the authors highlight how KP, a large healthcare system in the U.S., has addressed this public health issue and became the first carbon-neutral health system in the country.

## METHODS

This study employs an embedded, single-case study design to analyze KP’s decarbonization initiatives. The descriptive case study reviews the organizational strategies and outcomes of KP’s policies. The authors retrieved primary data from KP’s publicly available sustainability reports (2015–2023). To corroborate the findings, these documents were supplemented with peer-reviewed studies evaluating KP’s environmental interventions and validated by KP’s sustainability team through structured email correspondence.

## RESULTS

From its original founding nearly 75 years ago, a core tenet of KP has been health equity and inclusiveness, including an emphasis on social and environmental determinants of health. The organization initiated an on-site solar program and required medical product suppliers to complete sustainability scorecards.^16^ The on-site solar program was first introduced in 1980 at the Santa Clara Medical Center in Silicon Valley.^17^ This pioneered further investments into solar power, including California’s first renewable microgrid at the Richmond Medical Center.^18^ Currently, over 100 of the 700 plus KP hospitals have solar panels on site, with continued efforts to increase this number.^18^

On a national scale, KP has a long-standing history of collaborating with community-based organizations, advocacy groups, and local agencies to advance health equity. These collaborations often arose through joint initiatives with Medi-Cal, municipal health programs, and local advocacy organizations. For example, in the Northwest, KP worked with regional social service agencies and energy assistance programs to help Medicaid members receive air-conditioning units at no cost during extreme heat events.^19^ In 2023, KP Orange County distributed $2.8 billion to local organizations, including HIV service providers, food banks, and Federally Qualified Health Centers, to expand health services, nutritious meal programs, and supportive care for vulnerable populations.^20^

In 2012, KP adopted an ambitious strategy focused on reducing greenhouse emissions by 30% by 2020.^21^ This ambition was further highlighted in 2016 with a pledge to become fully carbon neutral in 2020.^21^ Endeavors to achieve that goal included scaling the on-site solar program to include over 100 locations, signing 20-year off-take agreements for new utility-scale wind and solar power generation, and purchasing carbon offsets with health cobenefits.^18^

**Figure 1:**
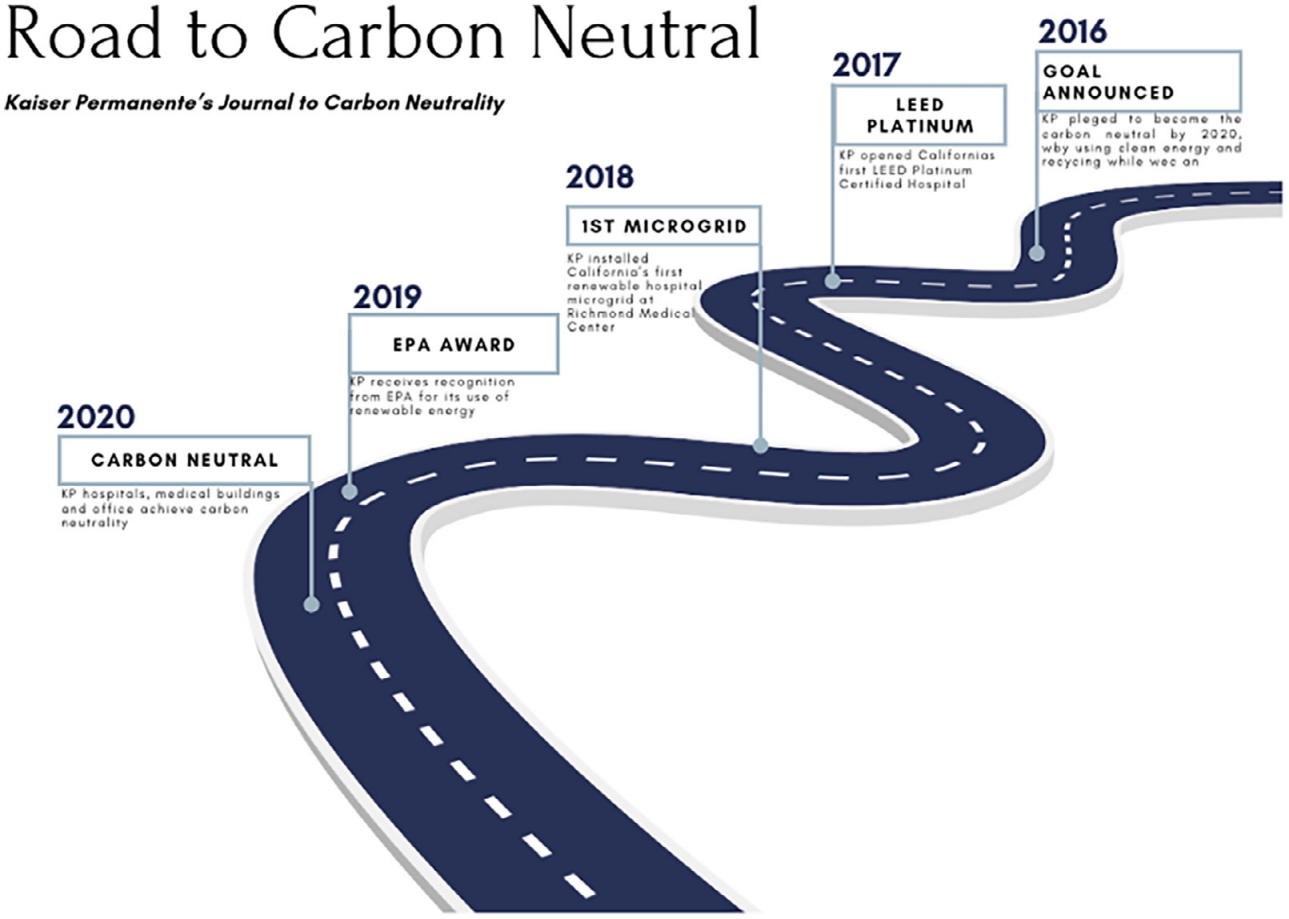
The 4 decarbonization pillars of collective action to further decarbonize.

**Figure 2:**
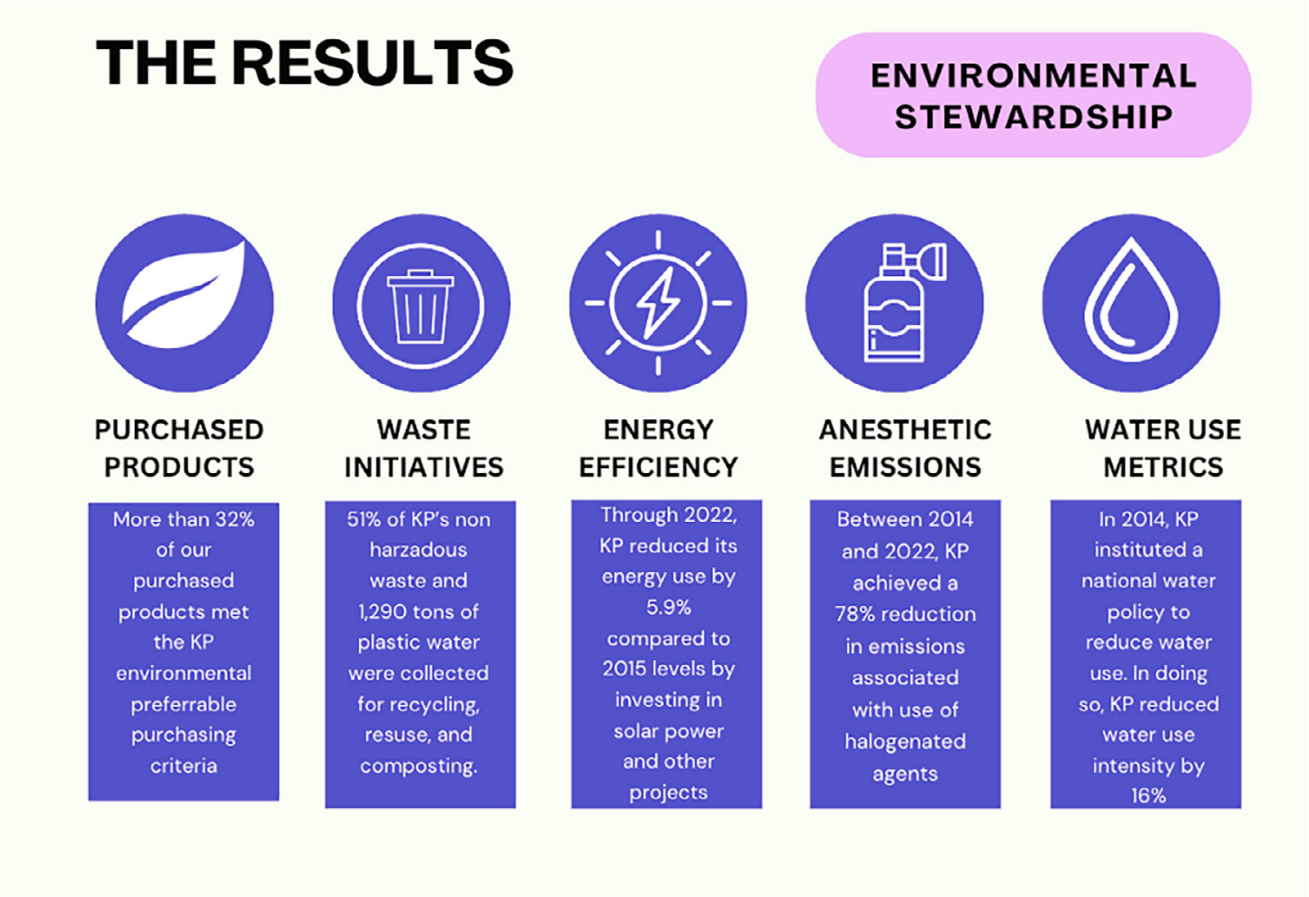
Results of Kaiser Permanente’s environmental efforts.

Other achievements include the KP San Diego Medical Center, the first Leadership in Energy and Environmental Design platinum–certified hospital, as well as the first renewable hospital microgrid at the Richmond Medical Center (Figure 1).^21^^,^^22^ With years of effort, KP was certified as becoming carbon neutral in 2020 and becoming the first health system in the U.S. to achieve this milestone.^18^ In addition, KP has improved energy efficiency by 6% since 2013, has decreased water use intensity by 22% since 2015, and has been recognized by the Environmental Protection Agency for its extensive efforts (Figure 2).^23^ As it stands currently, KP is the 8^th^ largest user of solar energy in the country.^24^

Future KP efforts are centered on cutting greenhouse emission by 50% by 2030 and aiming to achieve a net zero emission status by 2050.^25^ Recognizing that advancing efforts toward climate change mitigation and environmental stewardship necessitated collaborative engagement with its partners, KP and the Health Care Without Harm Group organized a roundtable involving hundreds of hospitals and medical device, equipment, service, and distribution companies in November 2022. The focus of this talk was addressing GHG emissions that would allow distribution companies to partake in decarbonization, especially considering that medical devices and supplies contribute to 7% of the U.S. health sector footprint.^16^ After this, the roundtable reconvened in April 2023, facilitated by Accenture, aimed to identify specific areas of collective action to decarbonize the healthcare distribution chain. These talks eventually resulted in a 24-month road map with 4 main areas of focus or decarbonization levers, which launched as a collaborative effort in April 2024.^16^

These 4 areas include renewable energy that would encourage companies to collectively commit to powering their operations with a certain percentage of renewable energy. Another avenue of interest was product innovation, which included reducing waste and introduction of more sustainable products on the market. Product utilization was another core tenet of the roundtable, which focused on limiting the use of single-use products and adoption of more sustainable workflows. Finally, the transportation aspect of this agreement centered on reducing transportation-related emission through development of algorithms to help with order consolidation, packing efficiency, and optimized delivery routes while avoiding last mile or overnight deliveries. With this proposal, the next steps involve establishing a formal collaborative effort to execute the 24-month road map with clear goals, membership criteria, and financial investment. Following best practices in governance, engagement, and accountability will be crucial for the success in further decarbonizing the healthcare system.

## DISCUSSION

KP’s decarbonization strategies mirror and extend approaches observed in other leading health systems. For instance, the National Health Service achieved a 30% reduction in emissions through centralized procurement of renewables, a tactic paralleled by KP’s 100% renewable electricity commitment.^4^ KP’s unique inclusion of supply chain partnerships with low-carbon vendors corresponds with some of the best practices documented by Health Care Without Harm.^25^ However, unlike European systems benefiting from national carbon pricing,^26^ KP’s progress relies heavily on voluntary processes, which underscores the need for national policy–enabled scalability.

Corporations such as Microsoft and IKEA have achieved carbon neutrality through similar strategies, including Marquis’s 2025 renewable energy investments^27^ and IKEA’s material reuse program.^28^ KP’s unique contribution lies in its integration of clinical practice with decarbonization. For example, KP developed medically tailored meal programs that simultaneously addressed food insecurity; educated patients on healthy-eating habits; and reduced emissions from food waste by serving plant forward, locally sourced meals, a dual benefit rarely prioritized by nonhealthcare entities.^29^ This suggests that healthcare systems may offer a model for decarbonization that remains unique to other sectors outside of healthcare but could be emulated.

From 2015 to 2025 period, KP navigated the evolving U.S. policy environments, from federal tax incentives for renewables to state-level mandates such as California’s SB 100 (2018) requiring 100% clean electricity. Although KP capitalized on these policies to accelerate solar procurement, the absence of a national carbon framework left it dependent on volatile state and regional programs. Notably, KP’s advocacy for the 2022 Inflation Reduction Act’s healthcare-specific grants^30^ demonstrates how healthcare systems can actively partake in policy building rather than just reacting.

### Limitations

Although this case study provides understanding into KP’s decarbonization strategies, several limitations must be considered. First, by relying on sustainability reports from KP (2015–2023), this could introduce self-reporting bias. The authors attempted to partially mitigate this issue by corroborating with other sources. Second, KP is an integrated healthcare system with unique financial and structural advantages, which could limit generalizability to other healthcare organizations. Third, the emissions data did not identify facility-level variations, limiting the analysis of KP’s policies on a site-to-site basis. Finally, although the 2015–2023 timeframe reviews the early adoption phases of KP’s endeavors, longer-term outcomes, such as the durability of supply chain reform, remain to be seen. These limitations emphasize the need for future studies focusing on cross-system comparisons to extend this study’s findings.

## CONCLUSIONS

The WHO declared climate change the most significant threat to human health. Healthcare systems must focus on climate initiatives to address this growing issue. KP's efforts to become carbon neutral highlight the importance of environmental stewardship on a systems level as well as practical means to achieve positive climate change effects.
